# Estrogen Receptors and Endometriosis

**DOI:** 10.3390/ijms21082815

**Published:** 2020-04-17

**Authors:** Elodie Chantalat, Marie-Cécile Valera, Charlotte Vaysse, Emmanuelle Noirrit, Mariam Rusidze, Ariane Weyl, Kelig Vergriete, Etienne Buscail, Philippe Lluel, Coralie Fontaine, Jean-François Arnal, Françoise Lenfant

**Affiliations:** 1 IUCT Oncopole, 31100 Toulouse, France; 2 INSERM-UPS UMR U1048, Institut des Maladies Métaboliques et Cardiovasculaires, Université de Toulouse, BP 84225, CEDEX 04, 31 432 Toulouse, France; 3 Department of Visceral Surgery, CHU Rangueil, 31400 Toulouse, France; 4 Urosphere, Rue des Satellites, 31400 Toulouse, France

**Keywords:** endometriosis, estrogen receptors, modulation, treatment strategy

## Abstract

Endometriosis is a frequent and chronic inflammatory disease with impacts on reproduction, health and quality of life. This disorder is highly estrogen-dependent and the purpose of hormonal treatments is to decrease the endogenous ovarian production of estrogens. High estrogen production is a consistently observed endocrine feature of endometriosis. mRNA and protein levels of estrogen receptors (ER) are different between a normal healthy endometrium and ectopic/eutopic endometrial lesions: endometriotic stromal cells express extraordinarily higher ERβ and significantly lower ERα levels compared with endometrial stromal cells. Aberrant epigenetic regulation such as DNA methylation in endometriotic cells is associated with the pathogenesis and development of endometriosis. Although there is a large body of data regarding ERs in endometriosis, our understanding of the roles of ERα and ERβ in the pathogenesis of endometriosis remains incomplete. The goal of this review is to provide an overview of the links between endometriosis, ERs and the recent advances of treatment strategies based on ERs modulation. We will also attempt to summarize the current understanding of the molecular and cellular mechanisms of action of ERs and how this could pave the way to new therapeutic strategies.

## 1. Introduction

**Figure 1:**
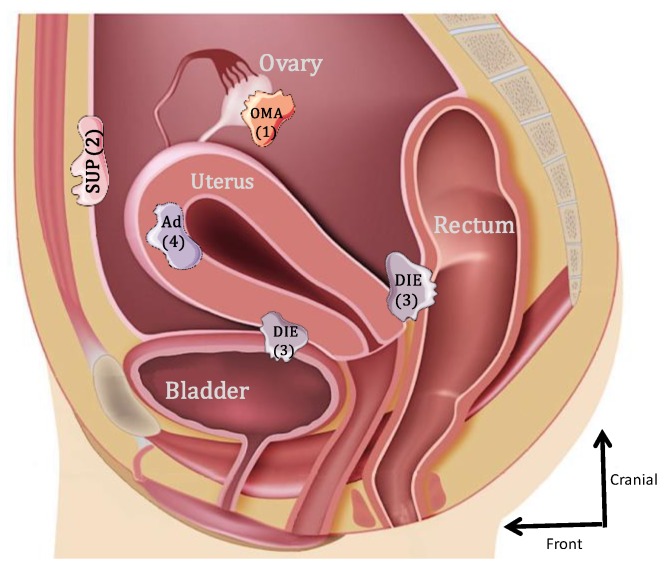
Schematic lateral view of the pelvis illustrating the 4 forms of endometriosis: 1: endometrioma (OMA); 2: superficial peritoneal endometriosis (SUP); 3: deep subperitoneal infiltrating endometriosis (DIE); 4: adenomyoma (Ad).

Endometriosis is one of the most frequently encountered benign gynecological diseases, known to occur in 6–10% of women of reproductive age [1,2]. It is an estrogen-dependent gynecological condition, defined as the presence and growth of endometrial-like tissue outside the uterine cavity [3]. Initially, three forms of endometriosis were classified according to their location: ovarian endometrioma (OMA), superficial peritoneal lesions (SUP) and deep subperitoneal infiltrating endometriosis (DIE) [4]. A fourth form that was frequently associated is internal endometriosis, since it is represented by the endometrium present within the myometrium (adenomyoma) (Figure 1). The most common locations for the ectopic endometrial implants are the ovaries, the fossa ovarica, the uterosacral ligaments, the posterior cul-de-sac, the rectum and sigmoid (20%) and more rarely in the pericardium, pleura and even the brain [5].

Endometriosis may give a wide array of symptoms ranging from pelvic pain (75% of cases) to catamenial pneumothorax but is mainly associated with severe and chronic pain, dysmenorrhea and deep dyspareunia as well as problems with fertility, although asymptomatic cases do arise [6]. The most evocative argument in favor of endometriosis is its cyclicity since it occurs very frequently during menstruation (catamenial pain) or in the peri-ovulatory period. Endometriosis is found in 25% to 40% of infertile patients and the risk of infertility is increased by 10 in case of endometriosis [7]. This infertility is often multifactorial including implantation disorders, pelvic adhesions and ovarian insufficiency, linked to the endometriomas altering the ovarian parenchyma. Endometriosis is therefore one of the most severe benign gynecological diseases because of its major consequences on fertility, daily quality of life with an alteration in sexual life and professional absenteeism [8,9,10]. 

Endometriosis is stratified by the American Society for Reproductive Medicine (ASRM) classification into four stages (I, II, III and IV) according to surgical evaluation of the size, location and severity of the endometriotic lesions and the occurrence of extensions of adhesions [11]. Other classifications, such as the European classification giving the FOATI score (Peritoneal focus-Endometrioma-Adherence-Tube-Inflammation), or the endometriosis fertility index (EFI) score, assess peritoneal inflammation as well as the progression of the disease and the rate of pregnancy obtained by in vitro fertilization after surgical treatment of endometriosis, respectively [12]. The definitive diagnosis for endometriosis is based on laparoscopy with biopsy followed by histological analysis. However, non-invasive diagnosis and markers of endometriosis that can confirm diagnosis are needed for treatment planning. Among imaging procedures, the magnetic resonance imaging (MRI) appears as the possibility to perform a complete assessment of all the pelvic compartments at one time. It therefore represents a good imaging technique for the preoperative staging of endometriosis [13].

Apart from the painful symptomatology of the disease, which is incapacitating [14,15], the morbidities of surgical management such as pelvic abscesses, rectovaginal fistulas and peritonitis participate in the alteration of quality of life [16]. For women with pain, surgery commonly provides temporary relief, although symptoms recur in up to 75% of women within two years, and further surgery is needed in many cases [1,17]. Medical treatment is often the first-line therapeutic option. Medical therapies historically have included combined oral contraceptives, progestagens, and agonists of gonadotropin-releasing hormone (GnRH), as well as androgens and non-hormonal treatments, such as painkillers and non-steroidal anti-inflammatory agents. Hormonal treatments for endometriosis focus on suppressing hormonal fluctuations (gonadotropin and ovarian hormones), resulting in the inhibition of ovulation and menstruation and a downstream decrease in inflammation. Hormonal treatment alone does not provide long-term disease control because it is often interrupted due to a significant amount of untoward side effects [18].

While the etiology of endometriosis still remains unclear, retrograde menstruation, in which uterine epithelial and stromal cells are disseminated and implanted into the peritoneal cavity *via* the fallopian tubes, is the most accepted mechanism for the pathogenesis of endometriosis. However, there is a missing link because the majority of women have retrograde menstruation (90% of women) but only 5% to 10% of women of reproductive age and 2.5% of postmenopausal women will develop lesions of endometriosis [1,19]. Moreover, retrograde menstruation does not explain the occurrence of endometriosis in extra pelvic locations. A second theory about the mechanism of the onset of endometriosis suggests that the epithelial peritoneal lining might transform into endometrial tissue under the influence of stimuli: this is the theory of coelomic metaplasia [20,21]. Another theory, of the benign lymphatic or haematogenous metastases, proposes an extraperitoneal dissemination of endometrial tissue *via* the lymphatic ducts and implies that the ectopic endometrial cells have migratory abilities [22]. Several risk factors including endocrine, genetic, biochemical, environmental, and immunological factors are effective in the initiation and progression of the disease [6,23]. These mechanisms might act in unison to cause endometriosis, but the main trophic factor in endometriosis is estrogen and estrogen exposure plays a crucial role in the development of the disease *via* estrogen receptors (ERs) [1].

The use of animal models in the study of endometriosis as well as clinical research have expanded our knowledge of pathogenesis and disease progression, highlighting the complexity of this disease that includes angiogenesis, inflammation, hormonal response and the associated signaling pathways. So, the aim of this review is focused on the role of ERs in the initiation and the progression of the disease. We also highlighted the latest advances of treatment strategies based on ERs modulation.

## 2. Levels of Estradiol and Estrogen Receptors in Endometriosis

**Figure 2:**
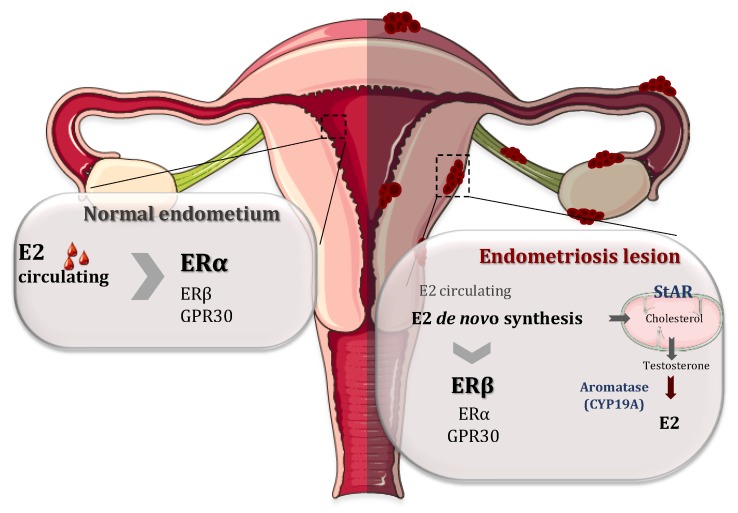
Respective roles of estrogen receptor α (ERα) and estrogen receptor β (ERβ) in the normal endometrium and endometriosis lesions. On the normal endometrium (left), 17β-estradiol (E2) coming from the circulation acts mainly on ERα while ERβ and G protein-coupled receptor 30 (GPR30) are less expressed. In contrast, in the endometrial lesions, ERβ expression is upregulated and the expression of ERα is attenuated. Moreover, there is a local accumulation of E2 mainly because the endometriotic lesions have the ability to synthetize E2 *de novo* from cholesterol, due to a higher expressions of steroidogenic acute regulatory protein (StAR) and CYP19A (aromatase), the two enzymes involved in the process of steroidogenesis.

It is well documented that endometriosis is intimately associated with steroid metabolism and associated pathways [1,24,25]. 17β-Estradiol (E2) is a key hormone for the growth and persistence of endometriotic tissue as well as the inflammation and pain associated with it. Estradiol reaches endometriosis by the circulation but it is mainly produced locally in the endometriotic tissue. This local estrogen accumulation has been considered to play an important role in the development and progression of endometriotic lesions by binding and activating ERs. This synthesis is upregulated in endometriotic tissue by altering the activities of enzymes involved in the biosynthesis and inactivation of estradiol [26,27]. In fact, endometriotic tissues have the ability to synthesize E2 *de novo* from cholesterol, because there is a high expression of two of the most important enzymes involved in the process of estrogen biosynthesis: aromatase (CYP19A1) and steroidogenic acute regulatory protein (StAR) (Figure 2). In contrast to endometriotic lesions, normal endometrium does not have the ability to synthesize estrogen due to the absence of these enzymes [27,28,29]. The enzyme aromatase is a member of the cytochrome P450 superfamily and is responsible for the last step in the synthesis of E2, i.e., the aromatization of androgens (androstenedione and testosterone) into estrogens (oestrone and E2, respectively). StAR facilitates the initial step of estrogen formation, the entry of cytosolic cholesterol into the mitochondrion. In addition, 17β-hydroxysteroid dehydrogenases (HSD17Bs) are involved in the formation of biologically active steroid hormones. The 17β-hydroxysteroid dehydrogenase 2 is implicated in the inactivation of E2 but the level and role of this enzyme are controversial [29,30].

The estrogen receptors (ERs) has two subtypes, estrogen receptors α and β (ERα and ERβ) encoded by estrogen receptor 1 (*ESR1*) and 2 (*ESR2*) genes, respectively. They belong to the nuclear receptor superfamily and exert biological functions in several ways [31]. In the classical genomic response, upon estrogen binding that leads to conformational changes, ERs dimerizes, translocates to the nucleus where they interact with the estrogen response elements or other transcription factors and recruit coactivators to modulate the transcription of target genes [31,32]. The ligand-induced transcriptional activity of ER involves the action of two distinct activation functions, i.e., AF-1 and AF-2 [33],. ERα can also activate non-nuclear signaling, which is also termed rapid/nongenomic/membrane-initiated steroid signaling (MISS) in a variety of cell types [31]. G protein-coupled receptor 30 (GPR30), a non-classical ER, can play a role in peculiar tissues and pathophysiological conditions [34,35,36].

In the normal endometrium, the expression of ERα, the primary mediator of the estrogenic action, is significantly higher than that of ERβ [37]. ERα and ERβ as well as c-myc, cyclin D1, and GREB1 mRNA expression levels were increased in ectopic tissue in comparison with both the normal and eutopic endometrium [38] and this predominant increase of ERα was found to be modulated according to the menstrual cycle [39]. *ESR2* mRNA levels were very low or nearly absent in the endometrial stromal cells [40,41]. On the other hand, in the ectopic endometrium from the cyst walls of ovarian endometriomas, *ESR1* mRNA and the expression of protein ERα were attenuated compared with eutopic endometrial tissues and cells, and in contrast, ERβ was upregulated [40,42,43,44]. *ESR2* mRNA levels were found to be 34-fold higher in endometriosis compared with the normal endometrium [40,43]. Elevated levels of ERβ existed in both nuclear and cytoplasmic locations in a mouse model of endometriosis [45].

The detailed mechanisms of the increase in ERβ remain unclear. The tissue- and cell-specific expression of a gene can be determined by DNA methylation. In fact, hypomethylation of the ERβ promoter region could be associated with the upregulation of the protein level in endometriotic tissues [40]. However, according to Maekawa *et al*., DNA methylation was not involved in the upregulation of *ESR2* [46]. In addition, the downregulation of *ESR1* in endometriosis could be caused by an aberrant DNA methylation of a specific region of the gene called the tissue-dependent and differentially-methylated-region (T-DMR) [46]. It has been hypothesized that *ESR2* suppressed *ESR1* expression in endometriotic cells in culture by binding to classical and nonclassical *cis*-regulatory elements in specific promoters of the *ESR1* [47]. DNA methylation seems to be an integral component of endometriosis, and according to Dyson et al. [48], the GATA family is a regulator of uterine physiology and aberrant DNA methylation in endometriotic cells correlates with a shift in GATA isoform expression that permits GATA6 expression in endometriosis instead of GATA2. This switch promotes the aberrant expression of many of the genes, including homeobox A10 (*HOXA10*), ERβ(*ESR2*), steroidogenic factor 1 (*NR5A1*), and aromatase (*CYP19A1*), which alter steroid signaling and responsiveness, and are critically involved in the disease development. In conclusion, epigenetic and genetic variations, such as post-translational modifications of ERs and coregulators, could alter their original function and become potent “drivers” of endometriosis progression [49].

## 3. The Role of ERs in Endometriosis

The molecular mechanisms regarding the specific contribution of each ER isoforms in the initiation and progression of the disease have been revealed in previous studies performed both in mouse models and in cells isolated from patients with endometriomas (summarized in Table 1). Beliard *et al*. reported a positive correlation between the proliferation and ER levels in normal and eutopic endometrium obtained from the peritoneum of women aged 26–40 years [50]. No correlation between apoptosis and estrogen receptor levels was found. However, the authors did not specify the antibody used and thus, did not differentiate between ERα and ERβ. Therefore, the ERα and ERβ knockout mice were used to surgically induce endometriosis-like lesion formations by injecting finely minced uterine tissue into the peritoneal cavity of the syngeneic host mice [51]. These mouse models revealed that both the ERα and the ERβ isoforms were required for the growth of endometriotic-like lesions [25,51]. However, the impact of estradiol which further increased the development of endometriosis-like lesions predominantly demonstrated the requirement of ERα for cell adhesion and proliferation, and for the neoangiogenesis that supports endometriosis-like lesion growth because the impact of an ERβ gene knockout was less than ERα gene deletion in the suppression of ectopic lesion growth [51]. More recently, the same mouse models were used to examine early disease development and its dependence on both E2 and ERα within 72 h of disease initiation. Using wild-type and ERα knockout mice as hosts or donors, the analysis of infiltrating cells after the initiation of endometriosis in mice, treated or not with E2, indicated a substantial infiltration of neutrophils and macrophages into the peritoneal cavity, irrespective of E2 or ERα status. However, IL-6 secretion was decreased 48 h after the disease initiation in αERKO to WT, as compared to WT to WT, providing evidence that E2/ERα/IL-6-mediated cross-talk played a partial role in increasing endometriosis lesion numbers [52]

Additionally, another study demonstrated that ERβ played a critical role in the development of endometriosis [53]. The modelization to surgically induce endometriosis lesions was slightly different, since the endometrial fragment isolated from the uterine tissue was sutured to the mesenteric membrane. They also used a mouse overexpressing ERβ and immortalized human endometrial epithelial cells injected into SCID mice. As a potential mechanism to evade immunosurveillance, they demonstrated that ERβ interacted with the apoptotic machinery in the cytoplasm to inhibit TNF-induced apoptosis, and with components of the cytoplasmic inflammasome, to increase IL-1β that contributes to cell survival, to enhance the cellular proliferation, invasion and the adhesion activities of immortalized human endometrial cells. In these models, ERβ also contributed to the epithelial–mesenchymal transition [53]. ERβ overexpression could then increase endometriosis-associated infertility by preventing the decidualization response in the stromal compartment of eutopic endometrium [53].

Using a new endometrium-specific FLAG-tagged human ERβ overexpression mouse model, the ERβ-transcriptomic and cistromic analyses demonstrated that ERβ stimulated the gene expression associated with IL6/JAK/STAT inhibitory signaling in ectopic lesions to enhance progression [54]. A genome-wide comparative analysis of ERβ-binding and gene expression in human endometriosis and endometrial tissues identified the Ras-like estrogen-regulated growth inhibitor (RERG) and serum and glucocorticoid-regulated kinase (SGK1) as key ERβ targets [44,55]. RERG induces ribosome biogenesis and the proliferation of primary endometriotic cells, thus integrating ERβ and prostaglandin E2 (PGE2). Signals at the RERG led to endometriotic cell proliferation [44]. Using siRNA knockdown of ERβ, the same group demonstrated that estradiol/ERβ also stimulated SGK1 expression and enzyme activity, leading to increased human endometriotic cell survival [55]. Finally, while steady state ERα:ERβ mRNA ratios were altered in stromal cells [56], the overexpression of ERβ in endometrial stromal cells significantly decreased ERα mRNA. This ERβ knock-down decreased the proliferation of endometrial stromal cells [47]. ERβ, acting as a suppressor of ERα, was then proposed to serve as a therapeutic target for endometriosis (see Part Treatments and Innovations).

In addition, the use of dominant negative mutants of estrogen receptor (DN-ER) genes, delivered to endometriosis cells (from ovarian endometriomas) via an adenovirus vector (Ad-DN-ER), abrogated the estrogen action on these cells and decreased cell proliferation, induced apoptosis and decreased cytokine production such as monocyte chemotactic protein-1, vascular endothelial growth factor, and interleukin-6 [57]. The invasion and migration of endometriosis eutopic stromal cells were regulated by estrogen/H19/miR-216a-5p/ACTA2 pathways. Specifically, the invasion and migration of these cells can be inhibited by the down regulation of H19 or ACTA2 [58].

One of the most characteristic pathogenetic features of endometriosis is the chronic pelvic inflammation. However, inflammation and estrogen production in endometriosis are linked by a positive feedback cycle in which the chronic overexpression of aromatase and COX2 supports the sustained production of estradiol and PGE2 in endometriotic tissue [59,60]. E2/ERβ stimulated PGE2 formation, whereas PGE2 stimulated estradiol synthesis [59,61]. Selective or nonselective COX inhibitors that disrupt PGE2 synthesis effectively reduced pelvic pain in endometriosis [62]. Moreover, in uterine microvascular endothelial cells, ERβ mediated estradiol-stimulated COX2 expression and PGE2 production [61].

New models such as three dimensional (3D) in vitro organoids have emerged to recapitulate the biological features of endometriosis. These in vitro organoids were initially developed from healthy mouse and human endometrium, expanded long-term and copied the phenotype of the epithelium in terms of response to hormones, including increased cell proliferation under estrogen and maturation upon progesterone [63,64]. Very recently, long-term expandable patient-derived organoids were prepared from endometrial disorders, including endometriosis and endometrial cancer [64,65]. They exhibited ERα and progesterone receptor (PR) expression as in the initial endometriotictissue. These organoids were further transplanted under the kidney capsule or into the peritoneum of NOD-SCID mice previously implanted with an estradiol pellet and were able to generate implants expressing the ER+ and PR+ lesions [65]. The expression of ERβ protein was not evaluated, and the large-scale transcriptomic analysis performed between the healthy endometrium and eutopic, or ectopic lesions did not reveal a differential expression of the *ESR2* gene, encoding ERβ, questioning the previous data showing the upregulation of ERβ [65].

**Figure 3:**
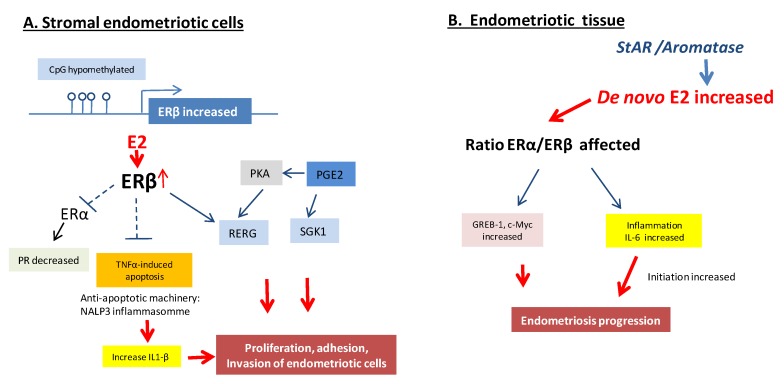
Molecular pathways of ER regulation in endometriosis lesions. (**A**). Overexpression of ERβ in the stromal endometriotic cells inhibits the TNFα-mediated apoptosis, acts as a suppressor of ERα, induces interleukin-1, co-stimulates Ras-related estrogen-regulated growth inhibitor (RERG) and serum and glucocorticoid-regulated kinase (SGK1) as key ERβ targets with co-stimulating prostaglandin E2 (PGE2) under the action of estradiol. (**B**). *De novo* increase of 17β-Estradiol (E2) in endometriosis lesions affecting the ratio of ERα and ERβ, impacting the inflammation and expression of some target genes such as *Greb-1* and *c-Myc* that results in endometriosis progression [2,27].

Altogether, it appears that ERβ and ERα act in a variety of ways to promote the proliferation of endometrial cells and tissue-invasion activity in endometriosis sites to establish ectopic lesions, with potentially a central role for ERβ in the development and pathophysiology of endometriosis (Figure 3). The overproduction of estradiol in endometriosis drives ERβ signaling to support endometriotic tissue survival and inflammation. Additionally, ERβ may have estradiol-independent pathologic actions. 

However, we must remain cautious about the role of ERβ in endometriosis. Andersson et al. questioned the expression of ERβ in several tissues due to the absence of validation of the anti-ERβ antibody used [66]. Moreover, the comparison of results obtained in different studies was hampered by the use of different methodologies and different endometriotic lesions (ovarian vs. peritoneal endometriosis).

Estrogens can also exert its effects through nongenomic signaling via cell membrane ERs. GPER (a seven-pass transmembrane G Protein-coupled Estrogen Receptor), also known as G protein-coupled receptor 30 (GPR30), has been identified as a novel receptor with binding ability to E2 in cell membranes, endoplasmic reticulum and the Golgi apparatus, and can trigger rapid estrogen non-genomic signaling independent of ERα and ERβ [31]. It can also regulate the rapid activation of the phosphatidylinositol 3-kinase (PI3K)/Akt and mitogen-activated protein kinase (MAPK) pathways. GPER expression in endometriotic tissues has been demonstrated to be relatively higher than in the normal endometrium and this induction was mediated by estrogen, stress and inflammation [67,68,69]. GPER is maximally expressed during the proliferative phase. In follicles of ovaries affected by endometriosis, GPER was found to be down-regulated, further supporting a role for GPER in folliculogenesis [69].

## 4. Treatments and Innovations in Clinical Management Related to ERs

The goals of medical therapy for endometriosis are pain control, improvement of the quality of life, prevention of disease recurrence, fertility preservation and the reduction of operative intervention [70]. Estrogen is the most hierarchically upstream and potent stimulus of survival and inflammation in eutopic and ectopic endometrial tissues. Thus, treatments for symptomatic endometriosis inhibiting ovarian estradiol production (contraceptive steroids, GnRH agonists, progestins, and aromatase inhibitors) would give limited benefit to women with autonomous endometriotic estradiol production [5]. In addition, the use of estrogen receptor ligands, inhibitors, and agonists also support the role of these receptors in endometriosis [24].

The potential of ERβ as a therapeutic target in endometriosis has been recognized. ERβ-selective compounds that act as estradiol antagonists in endometriotic tissue would be potential therapeutics. One study showed that a selective ERβ agonist (ERB-041) achieved lesion size regression compared with a vehicle in athymic nude mice implanted with fragments of normal human endometrium [71]. Another study treated surgically-induced endometriosis lesions in C57BL/6J mice with an ERβ-selective antagonist, such as PHTPP, to suppress ectopic lesion growth [53]. This discrepancy between the efficacy of an ERβ agonist or an ERβ antagonist can potentially be explained by difference in ERβ expression between normal and ectopic endometrial lesions, respectively in each study.

A link between ERβ, TNF and IL-1β has been highlighted in endometriosis lesions and would have a predominant role in endometriosis progression. Moreover, the blockage of TNF action using systemically administered recombinant TNF receptor type-1 or a monoclonal antibody against TNF prevented the establishment of endometriosis, or reduced the lesion size in a baboon model of endometriosis [72]. The SRC-1 isoform/ERβ complex played an essential role in the early stages of endometriosis pathogenesis and could be a next-generation endometriosis therapeutic target with reduced side effects compared to the current endometriosis treatment, because ERβ and the SRC-1 isoform have little expression in the eutopic endometrium [53]. In addition, estradiol induced COX2 via ERβ in endometriosis. The disruption of PGE2 synthesis via selective or nonselective COX inhibitors effectively reduced pelvic pain in endometriosis [73]. The two ER ligands (one highly selective ERβ ligand, the chloroindazole (CLI) and the ERα antagonist, oxabicycloheptene sulfonate (OBHS)) have strong ER-dependent anti-inflammatory effects on endometriosis lesions in vivo in a suture mouse model of endometriosis and in vitro, with primary human endometriotic stromal cells [25]. These ligands displayed potent antiestrogenic and anti-inflammatory activities mediated via the ERs in endometriotic cells.

Selective estrogen receptor modulators (SERMs) are synthetic molecules which bind to ERs and act either as antagonists or agonists, depending on the tissue type. The majority of findings were obtained in animal models and the effectiveness of SERMs in human endometriosis is still to be evaluated. In experimental models, SERMs showed a direct effect on endometrial blood vessels and suppressed endometrial prostaglandin production without the systemic effects of estrogen deprivation. Raloxifene, used for the treatment of postmenopausal osteoporosis, was tested in a rat model of endometriosis and was shown to have an estrogen-antagonist effect on the rat uterine tissue, producing implants’ regression [74]. In animal models, raloxifene showed comparable benefits with anastrozole in reducing the size of lesion [75]. In a randomized clinical trial, raloxifene statistically significantly shortened the time to the return of chronic pelvic pain [76]. Newer generation SERM, bazedoxifen, was being extensively studied for endometriosis therapy. In a mouse model, bazedoxifen, alone or combined with conjugated estrogen, reduced estrogen receptor expression in the endometrium and the size of endometriotic lesions [77,78]. Recently, Flores et al. have evaluated the effects of bazedoxifene paired with conjugated estrogens on reproductive hormones and uterine/ovarian appearance in premenopausal women. After one daily administration of bazedoxifene/conjugated estrogens for 12 weeks, all subjects demonstrated an LH surge without endometrial alterations or abnormal ovarian folliculogenesis [79]. Khine et al. evaluated the effects of SR-16234 on murine endometriosis-like lesions [80]. SR-16234 is a SERM which was reported to have ERα-antagonistic activity with a weak partially agonist activity to the ERβ receptor. They demonstrated that this SERM suppressed the growth and the expression of inflammation-associated genes in endometriosis-like lesions without inducing endometrial growth.

Moreover, estrogens can activate some non-genomic pathways of ERs, that activate rapid signaling between seconds or minutes, forming complexes with G proteins, growth factor receptors (IGF-1R, EGFR...); or non-receptor tyrosine kinase (e.g., SRC) that increase levels of nitric oxide, MAPK/ERK or PI3K/AKT kinases, and reactive oxygen species (ROS). These observations have prompted several groups to target these pathways, both in vitro and in vivo, testing the therapeutic potential of specific inhibitors of the MAPK/ERK, PI3K/AKT pathways [81].

Aromatase inhibitors offer an innovative approach to the treatment of this disorder, and these inhibitors, alone or in combination with more standard treatments, could be successful in eradicating treatment refractory endometriotic implants and improving pain symptoms when other medical therapies, such as GnRH agonists, have failed [1]. In premenopausal women, an aromatase inhibitor alone may induce ovarian folliculogenesis, and thus aromatase inhibitors are combined with a progestin, a combined oral contraceptive, or a GnRH agonist [82]. These combinations reduced visible lesions and pelvic pain refractory to other available medical and conservative surgical treatments [83,84]. In postmenopausal situations and in particular in cases of lesions that cannot be resected surgically, aromatase inhibitors are the treatment of choice [22,85].

Progesterone and progestins are also used in the management of symptomatic endometriosis. Selective progesterone receptor modulators (SPRMs) with primarily antiprogestogenic effects include mifepristone (RU486), asoprisnil, and ulipristal acetate [86]. They have demonstrated benefits in reducing pain and suppressing the extent of endometriotic lesions through several mechanisms: inducing anovulation, reducing the expression of aromatase, reducing the expression and enzyme activity of 17ß-HSD1 (hydroxysteroid dehydrogenase, which catalyzes the conversion of estrone to estradiol), altering ERs, inhibiting angiogenesis and decreasing the expression of matrix metalloproteinases needed for the growth of the endometriotic implant [87]. They are often called “mini-pill”, and it appears that this treatment should be the first-line therapy since they can eliminate pain and induce amenorrhea, improve the quality of life and reduce the size of endometriosis [88].

Apart from all these hormonal treatments, numerous immune-modulators or anti-angiogenic agents are currently being tested and developed [88,89] while the function of non-coding RNAs in endometrial physiology and physiopathology are also being discussed [90].

## 5. Conclusions

High estrogen production is a consistently observed feature of endometriosis and this review highlighted the fact that estrogen and its receptors play a key role in the pathophysiology of endometriosis. Targeting the local production of E2 might be a potential therapeutic strategy to block the development of endometrial disease. However, many questions remain opened concerning the specific role of ERα, which might be involved during the phase of initiation of the disease while it is necessary to confirm the upregulation of ERβ according to the anatomical site of the lesion after validation of the antibodies used. Moreover, although it is well known that endometriosis is a chronic inflammatory disease, and although some roles of ERα and ERβ have been mentioned in the inflammatory response, the impact of E2 and ERα/ERβ on immunity for the initiation and development of endometrial disease remain totally unresolved. The study of these new ER mechanisms with better diagnosis allowing to discriminate between a large variety of phenotypes should generate new ideas for the next generation of therapies for endometriosis that are clearly needed. They will be based on the development and improvement of current therapies, including oral GnRH antagonists, SERMs or SPRMs that will relieve pain symptoms, inflammation without suppressing ovulation. In this context, finding therapies that target both endocrine and inflammatory pathways might be of interest to better cure the origin of the disease.

## Figures and Tables

**Table 1:** Summary of the different studies on ERα and ERβ expression in endometriosis. IHC= Immunohistochemistry; IP= Immunoprecipitation. The “underline” is to emphasize the species: Human, mouse or rat and to also emphasize some cell types such as stromal or endothelial cells

Studies	ER or ERβ	Procedures	Models	Results
Enmark et al., 1997 [37]	ERβ/ERα	mRNA by RT-PCR	Rat tissues	Expression ERα was significantly higher than that of ERβ in normal endometrium.
Brandenberger et al., 1999 [56]	ERβ/ERα	mRNA by RT-PCR Southern blot Ligand-binding assays	Human normal endometrial and endometriosis-derived stromal cells	Ratio of ERα/ERβ mRNA in stromal cells were decreased in endometriosis as compared to normal endometrium
Fujimoto et al., 1999 [43]	ERβ and ERα	mRNA by RT-PCR IHC (Anti-ERα (MC-20) et ERβ –L-20) Southern blot	Human ovarian endometrioma Normal endometrium	In normal endometrium, ERα mRNA were expressed at a higher level than those of ERβ. However, ERβ mRNA expression was higher and over a much greater range in ovarian endometrioma than normal endometrium while ERα expression was lower and more random.
Matsuzaki et al., 2001 [39]	ERβ and ERα	mRNA RT-PCR assay TaqMan RT-PCR Nonradioactive in situ hybridization	Human ovarian endometrioma	The predominant expression of ERα in both glandular epithelial and stromal cells might have been essential for the development and growth of peritoneal and ovarian endometriosis The expression of ER was modulated according to the menstrual cycle
Beliard et al., 2004 [50]	-No differentiation between ERα and ERβ	Nuclear staining IHC Antibodies used not specified	Human endometriotic tissues (peritoneum)	-No correlation between apoptosis and estrogen receptor levels was found -A lower amount of steroid receptor was found in endometriotic tissues without cyclic modulation compared with the eutopic endometrium
Tamura et al., 2004 [61]	ERβ and ERα	mRNA and protein RT-PCR Western Blot	Human uterine microvascular endothelial cells	In uterine microvascular endothelial cells, ERβ mediated estradiol-stimulated COX2 expression and PGE2 production
Xue et al., 2007 [40]	ERβ and ERα	mRNA by RT-PCR Western blot:	Human endometrial and endometriotic stromal cells from ovarian endometriomas	-mRNA (34-fold) and protein levels of ERβ were higher in endometriotic stromal cells due to hypomethylation of a CpG island whereas level of ERα was lower in paired endometriotic versus endometrial stromal cells
Bukulmez et al., 2008 [42]	ERβ/ERα	mRNA and protein lIHC Histology qRT-PCR Western blot	Human endometriotic tissues	Expression ERβ is significantly higher than that of ERα in ectopic endometrium
Trukhacheva et al., 2009 [47]	ERβ and ERα	Si-RNA knockdown RT-PCR IP Western Blot	Human ovarian endometrioma	Overexpression of ERβ in endometriotic stromal cells significantly decreased ERα mRNA and protein levels, and ERβ knock-down significantly decreased proliferation of endometriotic stromal cells
Cheng et al., 2011 [45]	ERβ	mRNA by RT-PCR IHC Histology	Mouse: They transplanted steroid-manipulated, menstrual-like endometrium from K-ras(G12V/+)/Ah-Cre(+/+)/ROSA26R-LacZ(+/+)mice into gonad-intact immunocompetent wild-type mice	Elevated levels of ERβ existed in both nuclear and cytoplasmic locations in this mouse model of endometriosis
Burns et al., 2012 [51]	ERβ/ERα	mRNA by RT-PCR IHC	Mouse: Uterus samples injected in peritoneal cavity	ERβ gene knockout was less than ERα gene deletion in the suppression of ectopic lesion growth
Pellegrini et al., 2012 [38]	ERβ and ERα	mRNA byRT-PCR -IHC	Human endometrium with or without endometriosis	mRNA of ERβ and ERα were upregulated in the eutopic endometrial tissue of patients with endometriosis ERβ and ERα as well as c-myc, cyclin D1 mRNA expression levels were increased in ectopic tissue in comparison with both normal and eutopic endometrium
Monsivais et al., 2014 [44]	ERβ	Genome-wide comparative analysis of ERβ binding and gene expression	Human endometriosis and endometrial tissues	Ras-like estrogen-regulated growth inhibitor (RERG) and serum and glucocorticoid-regulated kinase (SGK1) are identified as key ERβ targets
Han and al., 2014 (Review) [49]	ERβ and ERα	Gene expression microarray data	Human endometriotic tissues	Aberrant levels of nuclear receptors and nuclear receptors co-regulators in ectopic endometriotic lesions were associated with the progression of endometriosis
Zhao et al., 2015 [25]	ERβ/ERα	mRNA by RT-PCR -IHC -Immunofluorescence	-Immunocompetent mice ERβKO and ERαKO -Human endometriotic stromal cells in culture	Both the ERα and the ERβ isoforms were required for the growth of endometriotic-like lesions
Han et al., 2015 [54]	ERβ	-IHC	They used mouse overexpressing ERβ and immortalized human endometrial epithelial cells injected in SCID mice	-ERβ also contributed to the epithelial–mesenchymal transition; ERβ overexpression could then increase endometriosis-associated infertility -ERβ played a critical role in endometriosis development, interacted with the apoptotic machinery in the cytoplasm to inhibit TNF-induced apoptosis and with the components of the cytoplasmic inflammasome to increase IL-1β
Monsivais et al., 2016 [55]	ERβ	siRNA knockdown of ERβ RT-PCR IHC Western Blot	Human ovarian endometriosis and normal endometrial tissues	Estradiol/ERβ also stimulated SGK1 expression and enzyme activity, leading to increased human endometriotic cell survival
Burns et al., 2018 [52]	ERα	mRNA by RT-PCR Flow cytometry Cytokine production	Mouse (WT, αERKO)	E2/ERα/IL-6-mediated cross-talk played a partial role in increasing endometriosis lesion numbers
Han et al., 2019 [53]	ERβ	ERβ-transcriptomic and cistromic analyses	New endometrium-specific FLAG-tagged human ERβ overexpression mouse model	ERβ stimulated gene expression associated with IL6/JAK/stat inhibitory signaling in ectopic lesions to enhance progression
